# Supplementary material for: A Proteomic Study of the Dual Oncogenic and Tumor-Suppressive Roles of SIRT3 in Lung and Breast Cancer Cell Lines
Source: Int J Mol Sci. 2026 Jan 28;27(3):1325. doi: 10.3390/ijms27031325 (PMC12898715; doi:10.3390/ijms27031325)
Supplement: Supplementary file 1 [file ijms-27-01325-s001.zip › ijms-4028917-supplementary.pdf]

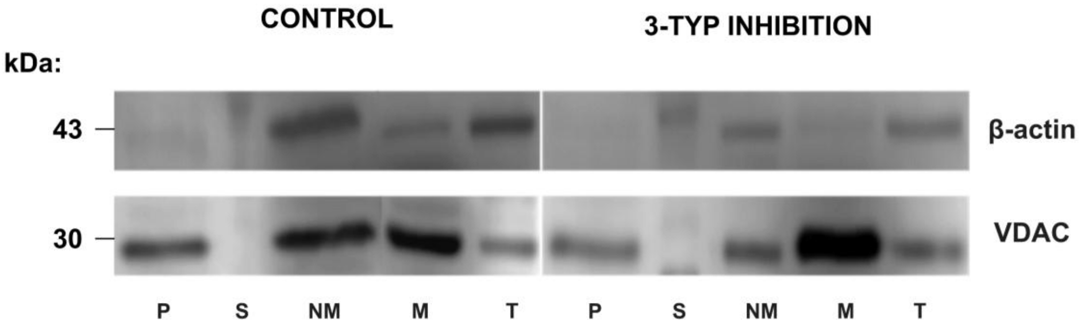

Figure S1. Evidence of mitochondrial enrichment in MCF-7 cells. *Immunoblot analysis of the different fractions obtained during mitochondrial enrichment, using β-actin as a cytosolic marker and VDAC as a mitochondrial marker: pellet (P), supernatant (S), non-mitochondrial fraction (NM), mitochondria (M), and total protein (T).*
